# Rapid and selective separation for mixed proteins with thiol functionalized magnetic nanoparticles

**DOI:** 10.1186/1556-276X-7-279

**Published:** 2012-05-31

**Authors:** Soo Youn Lee, Chi Young Ahn, Jiho Lee, Jin Hyung Lee, Jeong Ho Chang

**Affiliations:** 1 Bio-IT Convergence Center, Korea Institute of Ceramic Engineering and Technology, Seoul, 153-801, South Korea

**Keywords:** Silica-coated magnetic nanoparticles, Thiol, Mixed protein separation, BSA, LYZ

## Abstract

Thiol group functionalized silica-coated magnetic nanoparticles (Si-MNPs@SH) were synthesized for rapid and selective magnetic field-based separation of mixed proteins. The highest adsorption efficiencies of binary proteins, bovine serum albumin (BSA; 66 kDa; p*I* = 4.65) and lysozyme (LYZ; 14.3 kDa; p*I* = 11) were shown at the pH values corresponding to their own p*I* in the single-component protein. In the mixed protein, however, the adsorption performance of BSA and LYZ by Si-MNPs@SH was governed not only by pH but also by the molecular weight of each protein in the mixed protein.

## **Background**

The ability to separate and identify different proteins accurately and efficiently out of a biological sample is of utmost importance for application such as biomedicine, energy resources, environmental protection, and catalysis [1-3]. However, it remains imperative to understand the inherent physical characteristics of proteins, such as the isoelectric point (p*I*)^1^ and molecular weight. Because the technique is underlying the implicit assumption, the behavior of an intact biological system consisting of a plurality of proteins is coexistent mixed state [4].

In recent two decade, magnetic field-based separation using magnetic nanoparticles (MNPs) have received considerable attention because of their easy operation, uniqueness, and nano-scale size [5,6]. Besides their size-dependent properties, MNPs applications are affected by their surface functionalization. In this context, silica-coated MNPs (Si-MNPs) has several advantages arising from their stability under aqueous conditions (at least if the pH value is sufficiently low) and easy functionalization by hydroxyl group on the exposed silica surface surrounding MNPs [7,8]. A key consideration during protein separation process is to control the driving force of the sorbent surface behind the adsorption of proteins involves hydrophobic and electrostatic interactions [9]. Therefore, the successful adsorption of proteins onto MNPs depends on the proper surface modification.

Generally, thiol functional group (−SH) is known to play a significant role of cross-linking proteins, because the strongest nucleophile is present in the cystein thiol group of natural proteins [10,11]. Moreover, the value of the molecular electrostatic potential bond of thiol group may possess electronegative property [12]. Hence, it is presumed that thiol group can provide hydrophobic (non polar) condition to MNPs surface for improving protein adsorption [9]. To date, coupling biomolecules onto thiol-reactive surface has been achieved mainly for protein immobilization on gold-based biosensor surfaces through self-assembled monolayer system [13,14]. On the other hand, Si-MNPs are directly functionalized through the coupling of organosilanes (R′-Si(OR)^3^) including thiol group with free silanol groups of the silica surface [15]. Therefore, Si-MNPs having an easily modifiable silica surface as well as a superparamagnetic MNPs core might serve as an ideal platform for magnetic separation of proteins.

Here, we have exploited thiol group functionalized silica-coated magnetic nanoparticles (Si-MNPs@SH) as a magnetic sorbent for effective protein separation from aqueous condition. Furthermore, we discuss protein adsorption effects by Si-MNPs@SH in mixed proteins with various p*I* value and molecular weight, which are derived from straightforward experiments of two binary proteins: BSA and LYZ.

## **Methods**

### **Materials**

Silane coupling reagent were purchased from Aldrich Chemical Co. (St. Louis, MO, USA) and used as received. All other chemicals were of analytical reagent grade. BSA and LYZ were obtained from Sigma. Alexa Flour 488 and Texas Red fluorescence dyes were purchased from Molecular Probes Inc (Eugene, OR, USA). Distilled, deionized water was used for the preparation of all aqueous solutions.

### **Preparation of Si-MNPs and thiol group functionalization (Si-MNPs@SH)**

The silica-coated Fe_3_O_4_ magnetic nanoparticles were prepared using the method of Kang et al. [15]. The Si-MNPs were then modified successively with silane coupling agent to introduce the -SH. Briefly, 1 g of Si-MNPs was dispersed in 50 mL of anhydrous toluene containing 1 mg of (3-mercaptopropyl) triethoxysilane (MPTS), and the mixture was allowed to react at 110°C for 8 h under dry nitrogen. The resultants were separated by simple magnetic attraction and washed repeatedly with toluene and finally vacuum dried for further use.

### **Protein separation experiments**

First, adsorptions of proteins were carried out in 1 mL of protein solution dissolved in 100 mM phosphate buffer (pH 4.65, pH 7.5, and pH 11) and were added to disperse the Si-MNPs@SH (10 mg), according to the conventional method described elsewhere. The initial concentrations of BSA and LYZ solutions were both 5 μM. The reaction mixtures were incubated at room temperature for 10 min with continual agitation to the suspending particles. After removing the particles by magnetic attraction, the protein concentrations in the supernatants were determined by UV/VIS-spectrophotometer measurement at 278 nm. For the sequential separation of proteins from Si-MNPs@SH, the particles were suspended using 1 to 3 M NaCl and 0.1 M glycine solution (pH 2.3), which can desorb the proteins attached to the surface. After sharply stirring, the particles in the suspensions were separated with magnetic attraction, and the supernatants containing the desorbed proteins were collected for sodium dodecyl sulfate polyacrylamide gel electrophoresis (SDS-PAGE) analysis. The efficiency of protein adsorbed by the particles was calculated from the following formula:

(1) $$E_{ad}=(C_{i}-C_{f})/C_{i}^{*}\;100$$

where *E*_*ad*_ (%) is the efficiency of protein adsorbed by a unit mass of dry particles, *C*_*i*_ (μM) and *C*_*f*_ (μM) are the protein concentrations of the initial and final solutions, respectively. All the tests were conducted in triplicate.

### **Analysis of fluorescence-labeled proteins**

For the fluorescence labeling of BSA and LYZ, Alexa Flor 488 and Texas Red Protein Labeling Kit (Molecular Probes, UK) were used according to the manufacturer's instructions. Fifty microliters of 1 M sodium bicarbonate solution was added into a 0.5 mL of 100 μM BSA and LYZ solutions. The protein solutions were mixed with 0.5 mL of reactive Alexa Flor 488 and Texas Red dyes, respectively, for 1 h at room temperature. After the reaction, the remaining traces of free dyes were removed by column chromatography. In mixed protein solution, the adsorption of fluorescence-labeled BSA and LYZ to functionalized Si-MNPs was observed by confocal microscopy. Fluorescence spectra were measured using a microplate reader. The lasers provided excitation of Alexa Flour 488 for BSA and Texas Red for LYZ at 494 and 595 nm, respectively, and emitted fluorescent lights were detected at 519 and 615 nm, respectively.

### **Instrumentations**

The particle size and morphology of the Si-MNPs were determined by high resolution transmission electronic microscopy (HR-TEM) using a JEM-2000EX TEM (JEOL, Japan). The wide angle X-ray diffraction (XRD) patterns were taken with 40 kV, 160 mA Cu Kα radiation using a Rigaku Denki instrument. The magnetization of Si-MNPs@SH at room temperature up to 10 kOe was measured using a vibrating sample magnetometer (VSM 4179) (Oxford Instruments, UK). Identification and characterization of thiol functional group were carried out using X-ray photoelectron spectroscopy (XPS) (Sigma Probe equipped with monochromatic Al source, 15 kV and 100 W, Thermo Scientific, UK). Confocal microscopy was performed with a MultiProbe 2001 confocal scanning laser microscope, with an argon/krypton laser and ImageSpace Software from Molecular Dynamics, USA. The fluorescence intensity and absorbance of the samples were measured using microplate reader Infinite M200 (Tecan Ltd., Switzerland). The fluorescence was measured five times for each sample with a 20 μs integration time.

## **Results and discussion**

### **Synthesis and characterization of Si-MNPs@SH**

**Figure 1:**
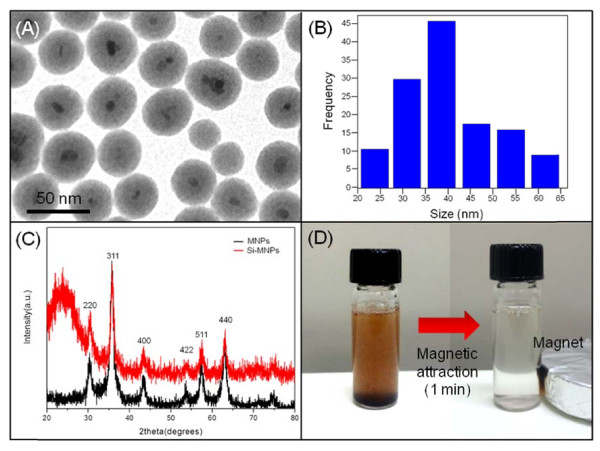
**Characterization, size distribution, XRD patterns, and magnetic attraction image of Si-MNPs.** (**A**) Characterization of Si-MNPs. TEM image shows the core-shell structured, (**B**) size distribution of Si-MNPs with 40 nm of average particle size (**C**) XRD patterns of MNPs and Si-MNPs, and (**D**) pictures showing the magnetic attraction for Si-MNPs.

The Fe_3_O_4_ magnetite core was synthesized via chemical precipitation of Fe^2+^ and Fe^3+^ salts (with a molar ration of 1:2) in a basic solution The Si-MNPs were directly produced by the sol–gel reaction of tetraethyl orthosilicate precursor with Fe_3_O_4_ magnetite [15,16]. The coating of silica shell onto Fe_3_O_4_ magnetite core provides a modifiable surface for the introduction of various functional groups for protein adsorption [17]. Figure 1A shows the generated Si-MNPs, which has well defined core/shell morphology and an average diameter of 40 nm (Figure 1B). In addition, the broad peak at around 2*θ* = 20° in the XRD pattern is due to the amorphous silica shell on the surface of the magnetite MNPs (Figure 1C). The diffraction peak at 220, 311, 400, 422, 511, and 440 are the characteristic peaks of magnetite nanoparticles (Figure 1C). Si-MNPs showed a saturation magnetization of 40 emu g^−1^ at 300 K (data not shown). The magnetic property of Si- nanoparticles such as gadolinum-based alloy nanoshell [18] or magnetite-based ferrofluid containing polymeric particles [19]. Moreover, it was observed that Si-MNP was dispersed in water by shaking or sonication and then easily separated with a magnet within 1 min (Figure 1D).

**Figure 2:**
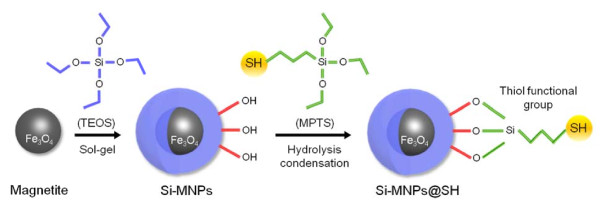
Scheme of thiol group functionalized Si-MNPs (Si-MNPs@SH) with MPTS.

**Figure 3:**
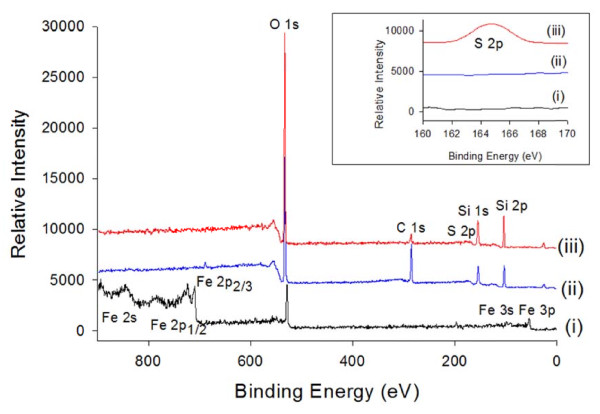
**X-ray photoelectron spectra ((i) Fe**_**3**_**O**_**4**_**magnetite, (ii) Si-MNPs, and (iii) Si-MNPs@SH).**

In this study, MPTS was used to modify the surface of the Si-MNPs to couple the thiol functional group with hydroxyl group of Si-MNPs surface (Si-MNPs@SH) via hydrolysis condensation reaction (Figure 2). Successful surface functionalization with MPTS was verified by XPS analysis (Figure 3). The spectrum data of both the Si-MNPs and Si-MNPs@SH showed that the Fe electronic configuration peaks were not detected, but Si peaks were detected at 149 and 100 eV for 2 s and 2p, respectively. The most prominent feature of the Si-MNPs@SH was the S electronic configuration peak that was detected at 165 eV for 2p. Consequently, the XPS data proved that thiol groups from MPTS were successfully coupled with the hydroxyl groups of the silica surface.

### **Separation of mixed proteins with si-MNPs@SH**

**Figure 4:**
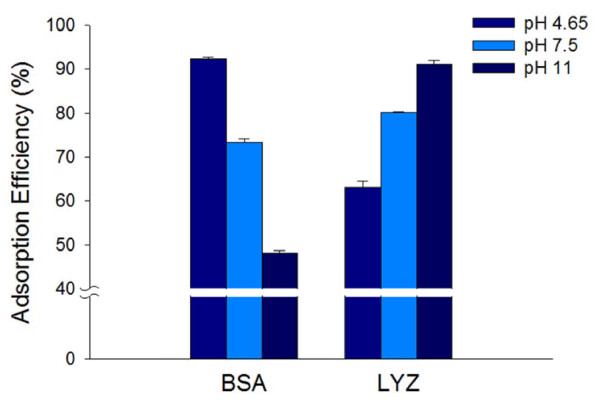
Adsorption efficiency of BSA and LYZ on the Si-MNPs@SH at pH 4.65, pH 7.5, and pH 11.

The conformation of proteins as a function of pH adsorbed at a surface is more compact when the pH is close to the p*I* of the native proteins [20]. This is due to the fact that proteins have a hydrophobic property with no net electric charges at the pH value corresponding with p*I*[9]. Hence, the Si-MNPs@SH can be introduced to investigate the effect of different pH ranges, that are pH 4.65, pH 7.5, and pH 11, on the adsorption of BSA (p*I* = 4.65) and LYZ (p*I* = 11) as a single-component solution [21]. As shown in Figure 4, the adsorption efficiencies of two proteins at the pH value corresponding to their own p*I* were more enhanced than opposite points. The adsorption efficiency of BSA at pH 4.65 (92.33 ± 0.34%) was 1.8 times higher than at pH 11 (48.09 ± 0.54%) but the adsorption efficiency of LYZ at pH 11 (91.21 ± 0.86%) was 1.5 times higher than at pH 4.65 (63.11 ± 1.32%). From our results, we notably observed that the high protein separation effects of both BSA at pH 4.65 and LYZ at pH 11 was by Si-MNPs@SH.

**Figure 5:**
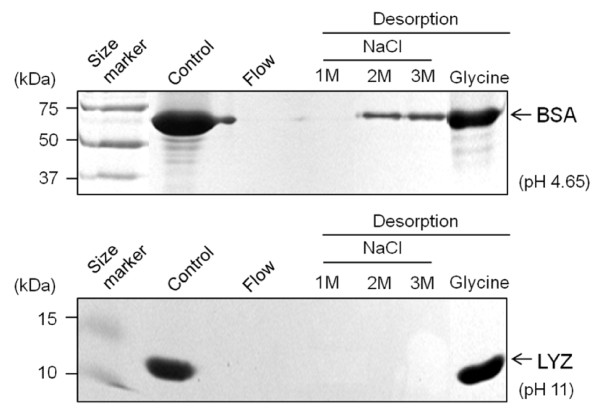
**The SDS-PAGE analysis of separated BSA and LYZ by the Si-MNPs@SH.** SDS-PAGE analysis of separated BSA (66 kDa) and LYZ (14.3 kDa) by the Si-MNPs@SH at pH 4.65 and pH 11, respectively. Initial protein (Control) and proteins desorbed from magnetic nanoparticles by 1 to 3 M NaCl and 0.1 M glycine solution (pH 2.3) were analyzed after protein incubation with Si-MNPs@SH for 10 min.

In general, the interaction of protein with other molecules is believed to be due to molecular electrostatic potential bond [12,13]. To obtain adsorbed proteins, thus, we also examined the degree of dissociation of BSA and LYZ from Si-MNPs@SH surface in the presence of a NaCl solution thorough electrostatic exclusion effect [22]. After the protein adsorption/desorption process, the protein patterns were observed by protein SDS-PAGE gel electrophoresis analysis (Figure 5). Unexpectedly, BSA and LYZ were not completely dissociated from Si-MNPs@SH even with the high NaCl molar concentration (3 M NaCl)**.** On the other hand, almost of the adsorbed proteins were estimated through desorption of the proteins from Si-MNPs@SH by 0.1 M glycine solution (pH 2.3) treatment. These data indicate that glycine may effectively attack disulfide links between proteins and thiol groups on the Si-MNPs@SH surface at acid condition [23].

**Figure 6:**
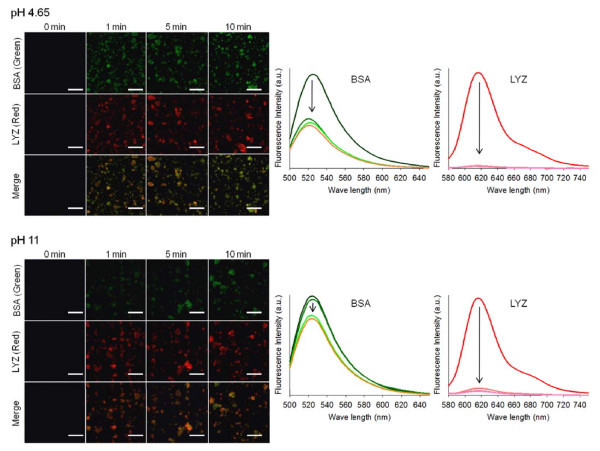
**Confocal images of BSA and LYZ adsorbed onto Si-MNPs@SH for 10 min.** Confocal images of BSA (green) and LYZ (red) adsorbed onto Si-MNPs@SH (the scale bar is 4 μm) for 10 min at pH 4.65 and pH 7.5 (left). Fluorescence intensities corresponding to the changes of emission intensity of protein solution (right).

In order to investigate the adsorption behavior of mixed proteins, identical mole ratios of BSA and LYZ in a binary mixed solution were adsorbed with Si-MNPs@SH. For observation with a confocal laser scanning microscope, BSA and LYZ were labeled with a green and red fluorescence dye, respectively. Figure 6 shows the microscope images and their quantified results for both BSA and LYZ molecules bound onto the Si-MNPs@SH surfaces, when coexisting in a binary mixed solution. Compared with the binding performance of two proteins in single-component solutions (Figure 4), the adsorption efficiency of BSA was just about 50% at pH 4.65 and barely 20% at pH 11. Meanwhile, the adsorption efficiency of LYZ was almost 100% at both pH 4.65 and pH 11. There are some clues as to why the binding effects of BSA and LYZ show such discrepancy between single-component and mixed proteins. First, structural stability of LYZ is higher than BSA under pH variation because LYZ is a highly helical, intrinsically stable and globular protein [24]. Second, proteins compete for adsorption on a weight and concentration basis. Therefore, from a mixed protein solution, protein adsorption occurs through a complex series of adsorption-displacement steps in which low-molecular-weight proteins (i.e., LYZ) that arrive first at a surface are displaced by relatively high-molecular-weight proteins (i.e., BSA) arriving later, until they are reached at a steady state [4].

## **Conclusions**

In conclusion, this study reported the fabrication of thiol group functionalized Si-MNPs (Si-MNPs@SH) and its use for effective protein separation in mixed proteins. For the purpose, binary proteins BSA and LYZ, which have different molecular weights and p*I* values, were used as model proteins for protein separation. Compared with the single-component proteins, the adsorption performance of BSA and LYZ by Si-MNPs@SH was governed not only by pH but also by the molecular weight of each protein in the mixed protein solution. The phenomenon was visualized by confocal microscope analysis of protein-bound Si-MNPs@SH. We expect that the results may have important implications in the binding effect of mixed proteins onto nano-sized particles and should also contribute to the further application of using magnetic nanoparticle-based technologies to separate proteins from intact biological samples such as blood plasma and serum.

## **Abbreviations**

pI, Isoelectric point; MNPs, Magnetic nanoparticles; -SH, Thiol functional group; Si-MNPs@SH, Thiol group functionalized silica-coated magnetic nanoparticles; BSA, Bovine serum albumin; LYZ, Lysozyme; MPTS, (3- mercaptopropyl) triethoxysilane; XPS, X-ray photoelectron spectroscopy.

## **Competing interests**

The authors declare that they have no competing interests.

## **Authors'contributions**

SYL carried out the biological and analytical studies. CYA and JL achieved the synthesis and characterization of the magnetic nanoparticles. JHL involved in instrumental characterization. JHC participated in the design of the study and coordination of the work as lead investigator. All authors contributed to the interpretation of the results and the drafting of the manuscript. All authors read and approved the final manuscript.
